# A Method for Characterising Human Intervertebral Disc Glycosaminoglycan Disaccharides using Liquid Chromatography-Mass Spectrometry with Multiple Reaction Monitoring

**DOI:** 10.22203/eCM.v035a09

**Published:** 2018-02-22

**Authors:** X. Liu, D. Krishnamoorthy, L. Lin, P. Xue, F. Zhang, L. Chi, R.J. Linhardt, J.C. Iatridis

**Affiliations:** 1 Departments of Chemistry and Chemical Biology, Biological Sciences, Biomedical Engineering and Chemical and Biological Engineering Center for Biotechnology and Interdisciplinary Studies, Rensselaer Polytechnic Institute, Troy, NY, USA; 2 Leni and Peter W. May Department of Orthopedics, Icahn School of Medicine at Mount Sinai, New York, NY, USA; 3 National Glycoengineering Research Center, Shandong University, Jinan, Shandong, China

**Keywords:** Intervertebral disc, disc degeneration, ageing, glycosaminoglycan, chondroitin sulphate, hyaluronic acid, heparan sulphate, mass spectrometry

## Abstract

Intervertebral disc (IVD) degeneration results in the depletion of proteoglycans and glycosaminoglycans (GAGs), which can lead to structural and mechanical loss of IVD function, ingrowth of nociceptive nerve fbres and eventually discogenic pain. Specifc GAG types as well as their disaccharide paterns can be predictive of disease and degeneration in several tissues but have not been comprehensively studied within the IVD. A highly sensitive mass spectrometry based technique with multiple reaction monitoring (MRM) was used to provide characterisation of chondroitin sulphate (CS), hyaluronic acid (HA), heparan sulphate (HS) and their disaccharide sulphation paterns across diferent anatomical regions of human IVDs. Principal component analysis further distinguished important regional variations and proposed potential ageing variations in GAG profles. CS was the GAG in greatest abundance in the IVD followed by HA and HS. Principal component analysis identifIed clear separation of GAG profiles between nucleus pulposus and annulus fibrosus in young and old specimens. Distinct patterns of predominantly expressed disaccharides of CS and HS between young and old IVD samples, provided preliminary evidence that important alterations in disaccharides occur within IVDs during ageing. This technique offered a novel approach to identify and quantify specific GAG disaccharides in human IVDs and the data presented were the first to offer insight into the spatial distribution as well as association with ageing of GAGs and GAG disaccharide sulphation patterns across the human IVD.

## Introduction

Low back and neck pain are the leading causes of global disability and among the top causes for absence from work (GBD 2015 Disease and Injury Incidence and Prevalence Collaborators, 2016; Hoy *et al*., 2010). The US economic costs for back and neck pain are approximately $253 billion for direct treatment and lost wages (Dieleman *et al*., 2016; Gunnar Anderson, 2014; Institute of Medicine (US) Committee on Advancing Pain Research, Care, and Education, 2011). Back and neck pain are strongly associated with intervertebral disc (IVD) degeneration. IVD degeneration correlates with the normal process of ageing but also involves structural failure that can arise due to external factors including genetics, environment, metabolic/hormonal changes and lifestyle (Adams and Roughley, 2006; Iatridis *et al*., 2009; Vo *et al*., 2016). The causes of pain and disability from IVD degeneration are complex and multifactorial involving biomechanical failure, extracellular matrix degradation, chronic inflammation and neurovascular growth into the normally avascular and aneural IVD (Freemont *et al*., 1997; Iatridis *et al*., 2013; Ito and Creemers, 2013; Johnson *et al*., 2015; Kadow *et al*., 2015; Mok *et al*., 2016; Molinos *et al*., 2015; Samartis *et al*., 2013).

IVD degeneration is nearly always characterised by degradation and depletion of the proteoglycan component of the extracellular matrix. Proteoglycans consist of proteins bearing covalently bound polysaccharides that belong to a family of sulphated glycosaminoglycans (GAG) including chondroitin sulphate (CS), keratan sulphate, dermatan sulphate, and heparan sulphate (HS). Hyaluronic acid (HA) is another GAG but is not synthesised on a protein core. Aggrecan is the predominant proteoglycan within the IVD, making up about 65 % of the nucleus pulposus (NP) and 15-20 % of the annulus fibrosus (AF) by dry weight (Gruber *et al*., 2011; Roughley, 2004). Aggrecan is also the most glycated proteoglycan, consisting mostly of CS and keratan sulphate chains, which form aggregates by interacting with HA (Sivan *et al*., 2014).

Proteoglycans serve many mechanical functions. The fixed charges of GAGs maintain IVD osmotic pressure and hydration to allow the IVD to withstand high compressive loads (Urban and McMullin, 1988). The loss of proteoglycans can directly result in decreased swelling and reduced water content in the IVD; and this loss of swelling pressure can lead to structural disorganisation, delamination, and annular fissures (Adams *et al*., 2000; Stokes and Iatridis, 2004). As a result, the total GAG content distribution in the IVD has been characterised with regional position, ageing and degeneration (Antoniou *et al*., 1996; Iatridis *et al*., 2007; Melrose *et al*., 2001; Roughley, 2006). Multiphasic finite element model predictions determine that GAG content and distribution are both important in defining IVD swelling pressure, hydration, mechanics and transport (Barthelemy *et al*., 2016; Gao *et al*., 2016; Iatridis *et al*., 2003; Jackson *et al*., 2011; Yao and Gu, 2006).

Proteoglycans also serve several biological functions through their interactions with cytokines, cell surface receptors, growth factors as well as other extracellular matrix proteins (Collin *et al*., 2016; Hayes *et al*., 2011). The ingrowth of nociceptive nerve fibres and blood vessels has been reported in painful, degenerated IVDs and is suggested to be a source of discogenic pain (Freemont *et al*., 2002). Interestingly, aggrecan is able to inhibit both endothelial cell adhesion/migration as well as the ingrowth of nerve fibres, and this effect is diminished following enzymatic degradation (Cornejo *et al*., 2015; Johnson *et al*., 2002; Johnson *et al*., 2005; Purmessur *et al*., 2015). Intact CS is identified as an essential contributor to the anti-angiogenic and anti-neuronal effects of notochordal cell conditioned medium making it an attractive target for inhibiting neurovascular ingrowth in degenerating IVDs (Cornejo *et al*., 2015; Purmessur *et al*., 2015).

The sulphation patterns of GAGs can also play important physical and biological roles (Rogers *et al*., 2011), yet these are surprisingly under-explored in musculoskeletal tissues. Specific patterns of human urinary CS and HS disaccharide sulphations are more predictive of the development/progression of renal dysfunction and hospital mortality than others (Schmidt *et al*., 2016). Similarly, age- and sex- related variations in CS sulphation in synovial fluid are known to exist (Nakayama *et al*., 2002). CS disaccharide sulphation also changes during maturation in ovine IVD (Collin *et al*., 2016). Cumulatively, these studies suggest that GAG disaccharides may change in IVD disease and degeneration. However, the GAG sulphation patterns in human IVDs and their functional changes in ageing and degeneration remain largely unknown motivating the development of new tools for their characterisation.

Most IVD GAG measurements use the dimethylmethylene blue (DMMB) assay and are limited to measurements of total GAG content. Only a few papers describe changes in GAG types and disaccharides in IVDs which use high-performance liquid chromatography (HPLC) or immuno-labelling of GAG epitopes (Collin *et al*., 2016; Hayes *et al*., 2011; Melrose *et al*., 2001; Nakayama *et al*., 2002; Sivan *et al*., 2014). It is notable that there is essentially no literature on GAG disaccharides in human IVDs, and methods previously used for their characterisation in IVDs of other species lack the sensitivity of liquid chromatography-mass spectrometry (LC-MS). The current study applied a, highly-sensitive method that combined the physical separation abilities of LC with the mass analysis abilities of MS, along with non-traditional statistical methods to isolate, identify and characterise GAG types and disaccharides in human IVDs with anatomical region. This project focused on methodological validation and characterisation of regional differences. We hypothesised that this novel GAG analytical technique would enable precise measurement of GAG disaccharides that would allow identification of even subtle differences in human IVDs across anatomical region and between specimens. Human IVDs ranged in age from 9-73 years old, with the majority of IVDs being a mature population with moderate degeneration and one very young and healthy sample (9 year old) for comparison (Table 1). Principal component analysis (PCA) was applied as a tool that utilised all the variances obtained from the GAG analyses to identify features that could find correlations in samples that were otherwise indistinguishable by traditional statistical analyses.

## Materials and methods

### Human tissue collection and preparation

**Fig. 1:**
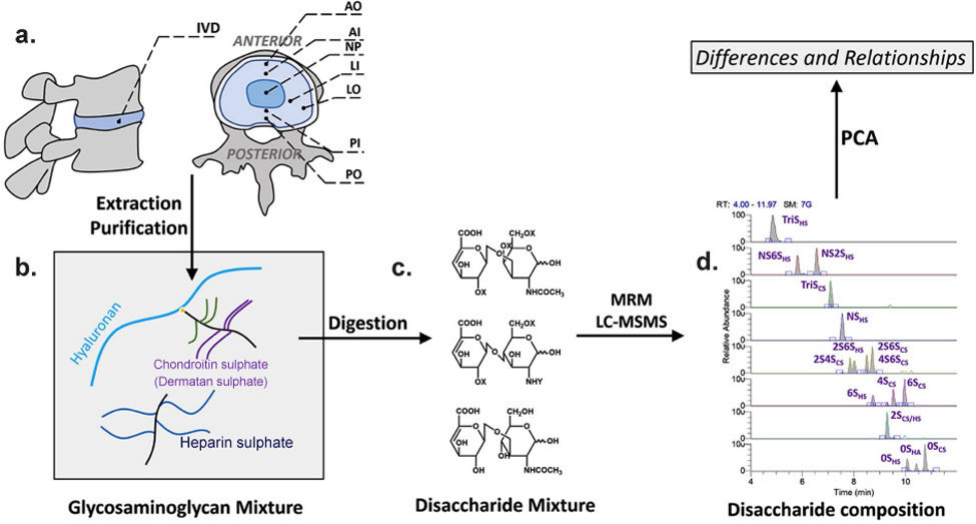
Flow chart for human intervertebral disc (IVD) specimen treatment for glycosaminoglycan disaccharide composition analyses. (**a**) 7 regions tested in each IVD: outer anterior annulus (AO), inner anterior annulus (AI), nucleus pulposus (NP), inner posterior annulus (PI), outer posterior annulus (PO), inner posterior-lateral annulus (LI), and outer posterior-lateral annulus (LO). (**b**) Glycosaminoglycans purified from the IVD regions, digested by the heparinases and chondroitinase ABC. (**c**) Structures of the disaccharides after adding heparinase I/II/III and chondroitinase ABC. ΔUA2X (1,3) GalNAc4X6X is generated from chondroitin sulphate (CS). ΔUA2X (1,4) GlcNY6X is generated from heparan sulphate (HS). ΔUA (1,3) GlcNAc is generated from hyaluronic acid (HA). X = H or SO_3_H. Y = H or SO_3_H or COCH_3_. (**d**) Extracted ion chromatograms (EICs) of the disaccharides.

Six totally intact human IVD samples were harvested from cadavers ranging in age from 9-73 years. Lumbar spines were obtained by autopsy services at Mount Sinai Hospital, with permission, and following procedures approved by the Institutional Review Board of Mount Sinai. The age, sex and cause of death of each donor were recorded (Table 1). IVDs were isolated from spinal specimens using a scalpel, and graded morphologically using the Thompson classification, which was modified for transverse sections (Acaroglu *et al*., 1995; Thompson *et al*., 1990). After cleaning and removing vertebrae, cartilage endplates and surrounding soft-tissues, each IVD was cut by scalpel into 7 anatomical regions: the nucleus pulposus (NP), posterior inner and outer annulus fibrosus (PI, PO), anterior inner and outer annulus fibrosus (AI, AO) and posterior-lateral inner and outer annulus fibrosus (LI, LO) (Fig. 1). A total of 156 specimens were analysed. 140 samples were obtained from 5 mature IVDs which had each of 7 anatomic regions divided into four equal sections for quadruple measurements. 16 samples were obtained from Hu29 (9 year old) IVD, which was smaller and consequently all 6 AF regions (AO, AI, PI, PI, LI, LO) were divided into two sections for duplicate measurements (12 samples) with sufficient NP tissue for quadruple measurements (4 samples). Wet weights were recorded.

The collected IVD samples were defatted with 0.5 mL acetone (Sigma-Aldrich, St. Louis, MO, USA) vortex for 30 min, and dried in the hood before digestion. The defatted samples were suspended in 0.85 mL of water with 0.15 mL of actinase E (5 mg/mL, Kaken Biochemicals, Tokyo, Japan) for proteolysis at 55 °C, until all the tissues were dissolved (36 h). Refer to Zhao *et al*. (2012) for validation and percentage recovery from the following GAG purification and desalting methods. GAGs were purified using Vivapure Q Mini H spin columns (Sartorious Stedim Biotec, Bohemia, NY, USA). Component eluted was then desalted by passing through a 3 kDa molecular weight cut off (MWCO) spin column (Millipore, Bedford, MA, USA) and washed twice with distilled water. The casing tubes were replaced, before 150 μL of digestion buffer (50 mM ammonium acetate containing 2 mM calcium chloride adjusted to pH 7.0, Fisher Scientific, Springfield, NJ, USA) was added to the filter unit. Recombinant heparin lyase I, II, III (pH 7.0-7.5) and recombinant chondroitin lyase ABC (10 mU each, pH 7.4) were added to each sample and mixed well (Su *et al*., 1996). The samples were all placed in a water bath at 37 °C for 12 h, after which enzymatic digestion was terminated by removing the enzymes through centrifugation. The filter unit was washed twice with 100 μL distilled water and the filtrates containing the disaccharide products were lyophilised.

The dried disaccharide samples were 2-aminoacridone (AMAC)-labelled (AMAC from Sigma-Aldrich, St. Louis, MO) by adding 10 *μL* of 0.1 M AMAC in dimethyl sulphoxide/acetic acid (17/3,V/V) incubating at room temperature for 10 min, followed by adding 10 μL of 1 M aqueous sodium cyanoborohydride (Sigma-Aldrich (St. Louis, MO) and incubating for 1 h at 45 °C. A mixture containing all 17 CS, HS and HA disaccharide standards (Iduron, Manchester, U.K., see Table 2 for structures) prepared at 6.25 ng/μL was similarly AMAC-labelled and used for each run as an external standard. After the AMAC-labelling reaction, the samples were centrifuged and each supernatant was recovered.

### LC-MS/MS analysis

The disaccharide analysis was performed as previously described (Sun *et al.,* 2015). LC was performed on an Agilent 1200 LC system at 45 °C using an Agilent Poroshell 120 ECC18 (2.7 μm, 3.0 × 50 mm) column. A triple quadruple mass spectrometry system equipped with an ESI source (Thermo Fisher Scientific, San Jose, CA) was used as the detector. The online MS analysis was done in the multiple reaction-monitoring (MRM) mode.

### Principal component analysis

PCA is an analytical method that uses multivariate data to differentiate between observations by transforming the data to a new coordinate system. The new coordinates, which are in *n*-dimension, are the principal components (PCs). The first coordinate, which provides the greatest variance, is called the first PC. The further PCs provide less variance than their preceding PCs. PCA can therefore be helpful in analysing high dimensional datasets *(i.e.* datasets with many sources of variation and output measurements) with dimensionality reduction that allow visualisation of PCs that highlight the greatest variance. Such methods were previously used to characterise complex datasets with large numbers of experimental variables characterising heparin (Liu *et al*., 2017; Zhao *et al*., 2015). Two sets of PCA were operated on software Origin 9.1 (OriginLab). A 2D PCA was performed to visualise all IVD regions and replicates to obtain an overview plot and to determine if GAG values were best distinguished by human subject (which vary by age), circumferential regional position or radial regional position. Percentages from total GAG composition, HS disaccharide composition and CS composition were chosen as variables. Two PCs were extracted where all the tested samples (156 total samples from 7 IVD regions) were observed. A 3D PCA was then performed to determine, with beter resolution, if GAG values were distinct by IVD regional position. In this second set of PCA, 15 variables were the HS, CS, and HA concentrations from total GAG composition of five patients (Hu29 was excluded because of its smaller number of replicates). Three PCs were extracted from the 15 variables. Seven different IVD regions, analysed in quadruple (biological replicates), were chosen as the observations.

### Statistical analysis

All values were represented as the mean ± standard deviation. One-Way ANOVA or Student's *t*-test was used to determine significant differences between regions and GAG type. The mature group consisted of 5 IVDs ranging in age from 47-73 years old. The young age group was represented by one IVD sample, age 9 years old. Average of all 6 samples together was also represented. Data were considered significant if *p* < 0.05.

## Results

### Distribution of CS, HA and HS GAG types across IVD regions

**Fig. 2:**
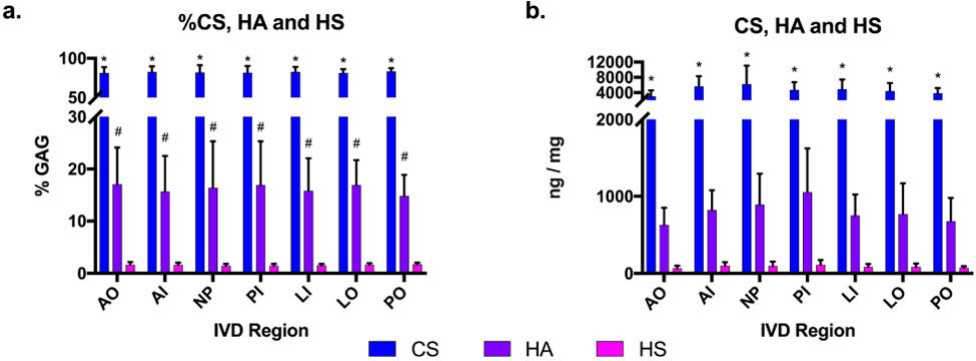
Composition of GAGs in different regions of the IVDs. (**a**) Percentage of each GAG type out of total GAG content. Percent of Chondroitin sulphate (CS), Hyaluronic acid (HA), and Heparan sulphate (HS) in the outer anterior annulus (AO), inner anterior annulus (AI), nucleus pulposus (NP), inner posterior annulus (PI), outer posterior annulus (PO), inner posterior-lateral annulus (LI), and outer posterior-lateral annulus (LO). (**b**) Total GAG composition quantified as ng GAG/mg of dry tissue weight. Data are averaged across all 6 IVDs (*n* = 6) for each anatomical region of interest. One-Way ANOVA between GAG types within each region shows significance *compared to HA and HS if *p* < 0.05 and ^#^compared to HS if *p* < 0.05. Mean ± SD.

Each of the three primary GAG types (CS, HA and HS) was averaged across 6 specimens, within each of the 7 regions analysed. Comparisons between GAG types within each region confirmed that CS was the most abundant GAG component followed by HA and HS in all regions analysed, whether calculated as percent composition or total dry-weight (Fig. 2). No significant regional differences in GAG composition were identified, although trends indicated approximately 50 % (*p* = 0.2) greater CS per dry weight in the NP and PI regions compared to the outer AF regions.

**Fig. 3:**
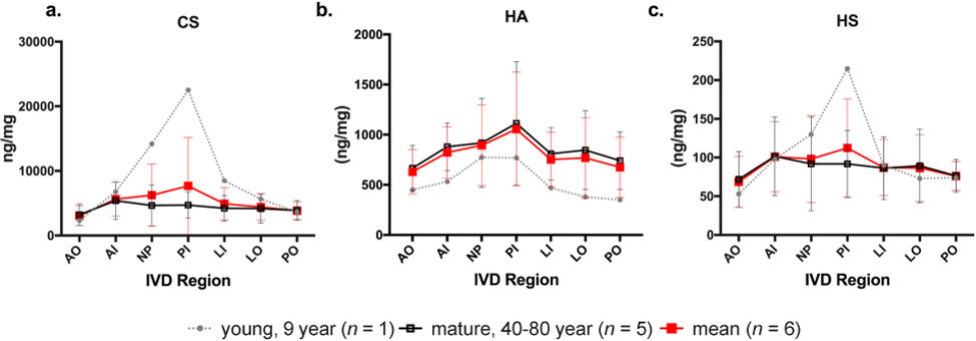
Concentrations of CS, HA and HS, in ng GAG/mg of dry tissue weight, across the 7 regions of the disc. The grey doted line represents the GAG composition of the young (9 year old) specimen *(n =* 1), mean of biological replicates for each region; the red line represents the average of all IVD specimens, mean ± SD *(n =* 6); the black solid line represents the average of all specimens except the one young (9 year old) IVD, mean ± SD *(n =* 5 mature specimens) for each region.

While the average GAG content of the 6 specimens together did not clearly show region specificity, separating mature specimens from immature ones more clearly revealed the variability in distribution of CS, HA and HS across regions of the IVD. Mature samples (age 40-80 years, *n* = 5) showed very little variability in CS and HS content, which was evenly distributed across all 7 regions (Fig. 3). HA content was greatest towards the middle IVD regions (NP + PI), with least values in the outer AF regions. In contrast, the young specimen, showed a very distinct GAG profile in relation to IVD region with substantially more CS and HS in the NP + PI regions and much smaller amounts in the outer AF (AO, PO, & LO). HA showed a similar pattern of increasing trend toward inner disc regions, yet HA content was less in all regions for the young than for the mature samples.

**Fig. 4:**
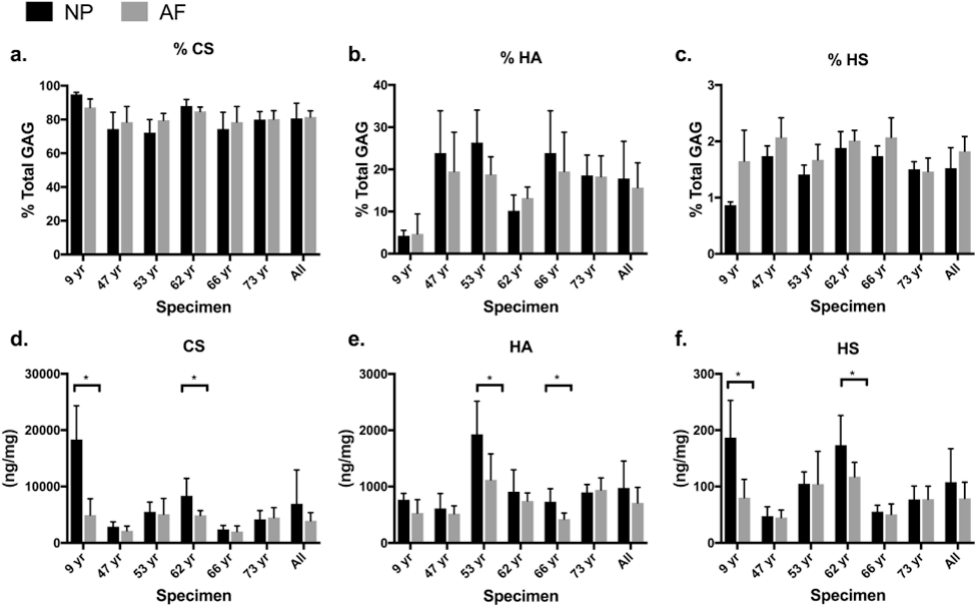
(**a-c**) Percentage of CS, HA and HS out of total GAG in the NP and outer AF regions (AO, PO and LO) in each of 6 IVD specimens. (**d**-**f**) Total CS, HA and HS represented as ng GAG/mg dry tissue weight in NP and outer AF regions (AO,PO,LO) in each IVD. For the 9 year old IVD, NP measurements are averaged across *n* = 4 biological replicates and AF measurements are averaged across *n* = 2 biological replicates. For all other IVDs NP and AF measurements are averaged across *n* = 4 biological replicates. Students *t*-test between NP and AF indicate significance if *p < 0.05. The average % GAG and GAG by dry weight of all 6 specimens is represented in the last two bars of each graph, labelled as “All”.

GAG values for AF and NP regions were compared to more prominently highlighted regional differences across specimens (Fig. 4). It was notable that the % CS, HA and HS did not change with anatomical region, highlighting that the % GAG is largely preserved with age and region. The GAG/dry weight was greatest in the NP and lowest in the outer AF regions, but this trend was not uniform across all specimens and GAG types. Notably, CS and HS had significantly more GAG/dry weight in the NP than AF only in samples with more total GAG content, suggesting that GAG is lost predominantly in the NP for most mature samples. HA GAG/dry weight was surprisingly not greatest in the samples with greatest total GAG content, but instead had largest values in the NP region of a 52 year-old subject, suggesting a patern that was distinct from ageing.

### Regional and age-related changes in CS and HS disaccharides

**Fig. 5:**
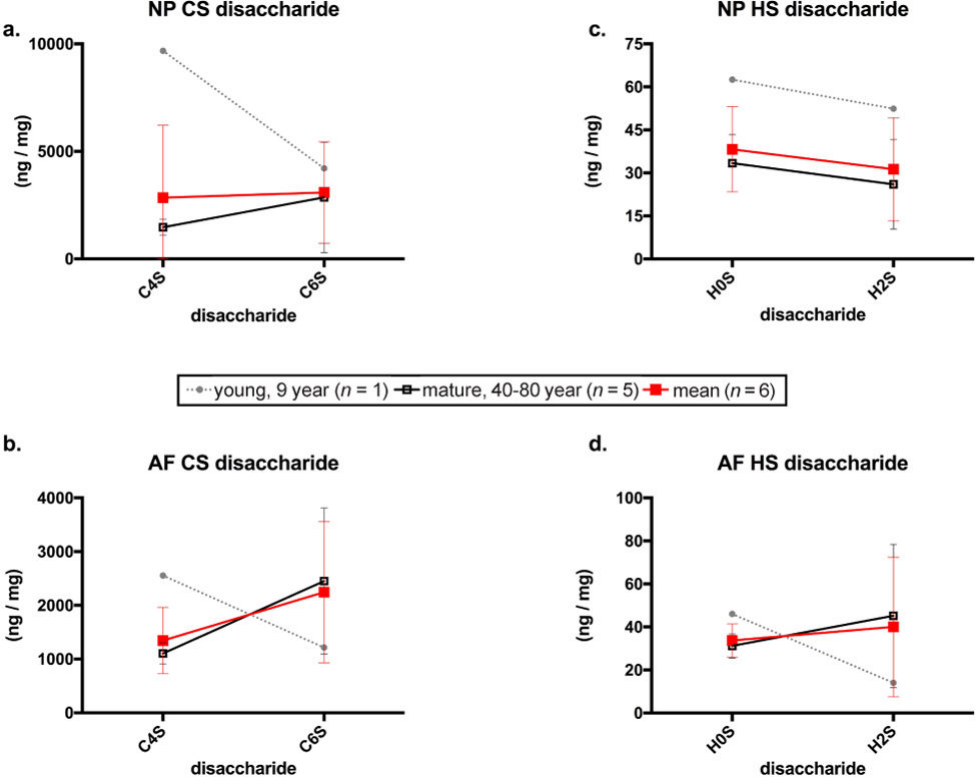
CS disaccharides, C4S and C6S, in the (**a**) NP and (**b**) AF. HS disaccharides, H0S and H2S, in the (**c**) NP (**d**) AF. Grey doted line represents the GAG composition of the young specimen, *n* = 1. Red line represents the average of all IVDs, *n* = 6. The black solid line represents the average of all IVDs except the young 9 year old IVD, *n* = 5. Data are represented as mean ± SD for the GAG content in ng GAG/mg dry tissue weight for the red and black lines. The grey doted line represents the average of the biological replicates for the one 9 year old IVD in the NP and AF.

Eight CS and HS disaccharides were quantified in the NP and AF of each disc. The data revealed two predominantly expressed disaccharides of each GAG type in both NP and AF: C4S, C6S, H0S and H2S. The distribution of these disaccharides within the NP and AF revealed region and potential age-related associations. While the concentrations of C4S and C6S were similar in NP (Fig. 5a), there was an almost 50 % greater amount of C6S compared to C4S in the AF (Fig. 5b), suggesting a change in sulphation position favouring C6S in human IVD AF. The C6S:C4S ratio also supported this trend (Table 3).

Separating the young specimen from the mature specimens exhibited potential age-related associations in CS sulphation patterns. In particular, the young specimen exhibited the greatest C4S in the NP and AF regions while the mature samples had 76 % (*p* = 0.06) more C6S than C4S in the AF (Fig. 5). It is also notable that while there was no difference in C4S content in the mature NP and AF regions, there was also the least amount of variance compared to that of C6S.

HS disaccharide content showed similar, but more subtle, differences within the NP and AF and between the young and mature specimens when examining closely the two predominant HS disaccharides, H0S and H2S. The mean HS disaccharide content of all six IVDs and only the five mature IVDs exhibited no notable differences in H0S and H2S in either the NP or AF regions. However, there was substantially more H0S and H2S in the NP of the young sample than in mature IVDs.

### Principal component analysis

**Fig. 6:**
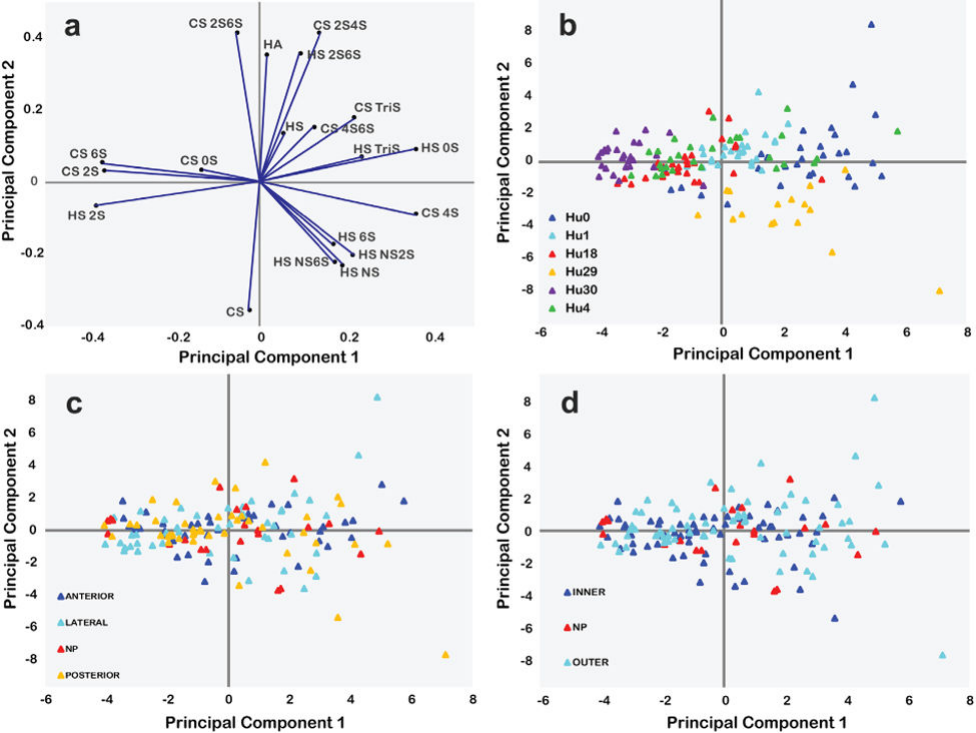
Principal component analysis (PCA) of all the IVD samples from different patients based on their total GAG composition, HS disaccharide composition and CS disaccharide composition. (**a**) PCA loadings indicating the coefficients of principal component (PC) 1 and PC2 for the GAG components and the disaccharide components. (**b**) PCA scores of the IVD samples on principal component (PC) 1 and PC2. Samples were grouped by patients. (**c**) PCA scores of the IVD samples on PC 1 and PC2. Samples were grouped for different regions by colour considering anterior / lateral / posterior AF regions or nucleus pulposus (NP) regions. (**d**) PCA scores of the IVD samples on PC 1 and PC2, samples were grouped by inner / outer AF regions or the NP regions.

GAG composition and disaccharides showed limited statistical variations with age or region, using traditional statistical analyses (Fig. 2-5). Principal component analysis (PCA) was then applied, as a tool that utilises all the variances obtained from the MRM LC-MS analyses, to identify structural features that could be used to find correlations between the IVD samples based on human subject and anatomical regions that were difficult to identify using traditional statistical analyses. Two PCs were chosen for the first set of PCA, which input all the disaccharide compositional information as either the input variables (total GAG, HS disaccharide and CS disaccharide compositions) or included all the IVD samples as individual observations, without any assignments. The loading plot, Fig. 6a, revealed which of the input variables were influential. For example, the inner anterior annulus fibrosus sample NO.1 from patient Hu0, located at [(PC1, -0.663), (PC2, -2.028) ], suggested dominant variables with negative PC1 coefficient values, such as (the percentage of CS in total GAG composition, the percentage of 2S in HS disaccharide composition, the percentages of 2S6S, 6S, 2S, 0S in CS disaccharide composition) and dominant variables with negative PC2 coefficient values, such as the percentage of CS in total GAG composition, the percentage of NS6S, NS2S, NS, 6S, 2S in HS disaccharide composition, the percentages of 4S in CS disaccharide composition, were at larger amounts in the GAG composition or disaccharide composition. Among all these variables, ones with both negative PC1 coefficient values and negative PC2 coefficient values, such as the percentage of 2S in HS disaccharide composition and the percentage of CS in the total GAG composition, were at the largest amounts. The score value plots (Fig. 6b,c,d), gave the amounts of the PCs in each of the different observations. PC1 and PC2 were the variable combinations of the percentage composition of the disaccharide components and the GAG components. As a result, each IVD sample had its PCA scores related to its GAG compositional information. In the initial steps of the PCA, all the IVD samples were analysed without any assignments. After the scores were output as 156 unlabelled points, the points were grouped based on different patients. IVD samples from each patient were labelled a different colour (Fig. 6b). Samples were then grouped for different regions by colour, considering anterior / lateral / posterior AF regions or nucleus pulposus (NP) regions (Fig. 6c), or grouped by inner / outer AF regions or the NP regions (Fig. 6d). IVD samples from identical patients clustered together in the score value plot (Fig. 6b) showing both PC1 and PC2 values were not far from each other. For example, IVD samples from Hu30 all had negative PC1 values and PC2 values around 0, which indicated that samples from Hu30, 6S, 2S, and 0S were at larger amounts in CS disaccharide composition and 2S was at larger amount in HS disaccharide composition. Similarly, IVD samples from patient Hu29 had positive PC1 values and negative PC2 values, which indicated that 4S_CS_ and 6S_HS_, NS_HS_, NS2S_HS_, NS6S_HS_ were at larger amounts. The two most distinguishable subjects, Hu30 and Hu29, were the oldest and youngest among all 6 IVD samples, suggesting important ageing effects on IVD GAG composition, even though the other samples overlapped. IVD regions were not distinguishable using 2 PCs by the anterior / lateral / posterior AF regions or the nucleus pulposus (NP) regions, and the inner / outer AF regions or the NP regions. By comparing the 3 kinds of different groupings (Fig. 6b,c,d), it was concluded that IVD samples were more distinguishable on different human beings than on different IVD regions.

**Fig. 7:**
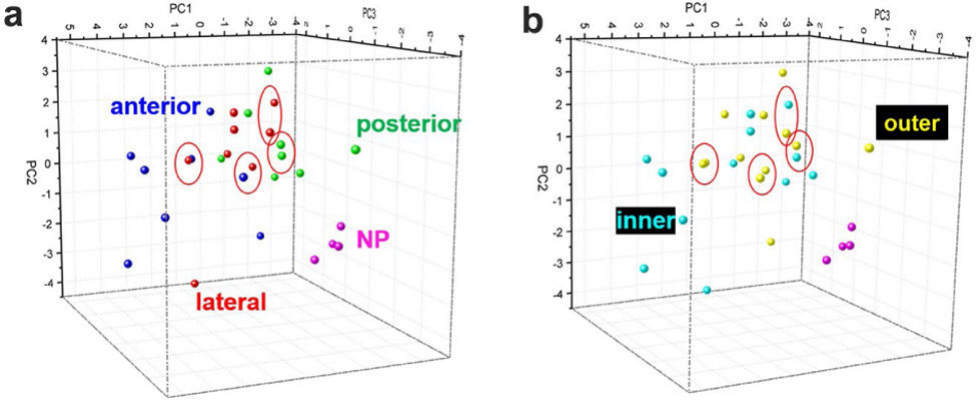
Principal component analysis (PCA) of the IVD samples from different IVD regions based on the total GAG compositions. Panel (**a)** and (**b)** are the same set of results distinguished by different groups. (**a**) Posterior, anterior and lateral AF regions were highlighted. (**b**) Inner and outer AF regions were highlighted.

Identification of IVD regions, based on GAG composition, was successful by using three PCs on a second set of PCA to increase resolution of regional differences. The GAG composition differences caused by differences between patients were neglected by reconstructing variables from different patients together (Table 4). Samples from anterior, posterior, lateral AF regions or NP regions were labelled in different colours, (Fig. 7a) samples from inner, outer AF regions or NP regions were also labelled in different colours (Fig. 7b). The coefficient values of the 15 variables (Table 4) and the scores of the 28 observations (Table 5) provided each data point with its PCA scores, related to its GAG compositional information to generate two 3D plots (Fig. 7a,b). Using three PCs IVD samples from NP regions were clearly identifiable from AF regions. Furthermore, the AF regions were better resolved as compared with the 2D plots using 2 PCs. However, some crosses between different AF regions were observed (red circles). Comparing the groupings in the 3D plots, we could conclude that they got similar PC scores because they were either from the same anterior/posterior/lateral regions or the same inner/outer regions. However, both analyses distinguished NP regions from other AF regions.

## Discussion

Proteoglycans play a crucial role in IVDs, by maintaining healthy biomechanical behaviours and performing bioactive functions including the inhibition of neurovascular ingrowth that can cause painful conditions (Adams and Roughley, 2006; Iatridis *et al*., 2013; Purmessur *et al*., 2013; Vo *et al*., 2016). About 90 % of the molecular mass of proteoglycans, including aggrecan, consists of GAGs so that most proteoglycan analyses in the IVD focus on total GAG content. However, GAG types and their disaccharide sequences are also important since they affect interactions with other macromolecules that influence multiple biological processes (Kiani *et al*., 2002). They can also predict the progression or development of disease and degeneration in various tissues (Collin *et al*., 2016; Schmidt *et al*., 2016; Uesaka *et al*., 2002). The current study characterised, for the first time, GAG types and disaccharides in human IVDs using a sophisticated and sensitive mass spectrometry based technique. IVD specimens ranged from 9-73 years old, and each IVD was analysed in 7 distinct regions that included the NP as well as inner and outer portions of the AF in the anterior, posterior and lateral regions. The focus was to use LC-MS with MRM and PCA to identify variances associated with different anatomical regions in the human IVD. Most IVDs were moderately degenerated from mature humans (47-73 years old), although a single sample with Thompson grade 1 (9 years old) was included to highlight potential differences with degeneration and ageing.

Analysis of GAG types showed CS as the most abundant GAG, followed by HA and HS in all IVD regions. Most of these mature IVD samples had similar amounts of CS, HA and HS in the NP and AF, although differences in the composition within the NP compared to AF regions was most evident in the young specimen as compared to the mature specimens. These data were generally consistent with literature that show greatest GAG content in the NP region and inner AF samples, particularly for young samples (Antoniou *et al*., 1996; Iatridis *et al*., 2007). Antoniou *et al*. also report signifficantly greater amounts of GAG in the NP than outer AF regions for older IVD specimens (Antoniou *et al*., 1996), in slight contrast to data reported here. It is likely that the less prominent regional differences found were due to the use of older subjects and the use of larger tissue samples that encompassed a greater radial variation than other data sets (Antoniou *et al*., 1996; Iatridis *et al*., 2007). Even with our less prominent regional variations, PCA was demonstrated to be a powerful tool capable of strong distinction of GAG profiles between NP and AF regions. Furthermore, PCA also allowed relatively good distinction of GAG profiles from different human specimens. An interesting regional observation was that multiple GAG types had greater content in PI than NP regions, which could only partly be explained by the inclusion of some NP tissue in the large PI samples. The lower value of GAG content in NP than PI region for multiple measurements in the current study was consistent with heterogeneities previously observed in the mature IVD samples. Specifically, a small ‘dip’ in total GAG is observed in the central NP region of multiple mature human IVD samples, using DMMB and fixed charge density measurements, and is also noted as an intranuclear cleft with reduced T2 values on MRI (Antoniou *et al*., 1996; Haughton, 2006; Iatridis *et al*., 2007).

The mass-spectrometry-based method used is reported as being applied to investigate GAGs in human urine samples and validated using comparisons with DMMB (Schmidt *et al*., 2016; Sun *et al*., 2015; Yang *et al*., 2017). Total GAG as measured using LC-MS is known to correlate with that quantified using DMMB, but the relationship is nonlinear (Schmidt *et al*., 2016). GAG concentrations reported with the DMMB method are greater than for the mass spectrometry method used in the current study, since the DMMB assay measures total GAG and can also detect other polyanions, such as DNA or mucins, resulting in greater background. The mass spectrometry method, used in the current study, relied on multiple reaction monitoring (MRM) mass spectrometry, which was selective and ultra-sensitive. This method was based on disaccharide analysis and used chondroitinase ABC and heparinases so it could only detect CS/DS, HS, and HA building blocks. Not all disaccharides were fully recovered from purification steps and KS was also not detected which also contributed to lower concentrations than expected from DMMB. There were sufficient similarities between our data and the literature to validate this mass spectrometry approach, for characterising GAG content in human IVDs, with our suggestion that total DMMB is a simpler method when only total GAG are needed. LC-MS is a more involved method offering specificity of information to characterise GAG types and disaccharides. Furthermore, caution must be exercised when comparing total GAG amounts across methods.

LC-MS was also capable of assessing multiple specific CS and HS disaccharides across different IVD regions and between specimens. Of the 17 CS disaccharides measured, C4S and C6S were found in greatest abundance. C4S and C6S are implicated in inhibiting neurite growth through their presence in NP notochordal cell conditioned media (Purmessur *et al*., 2015). Furthermore, increases in the C6S:C4S ratio measured in other tissues such as articular cartilage and synovial fluid is considered a strong indicator of joint ageing (Hickery *et al*., 2003; Nakayama *et al*., 2002). While the NP generally exhibited similar amounts of each disaccharide, the AF exhibited more C6S than C4S. Interestingly, the young IVD had more C4S than C6S in both NP and AF regions while mature IVDs had more C6S than C4S. Similarly, the C6S:C4S ratio in both NP and AF of the mature human IVDs were increased compared to that of the young. These data suggested a potential shift in CS sulphation with age favouring that of C6S. Notably, a similar inversion of sulphation pattern is observed in another study using bovine IVDs, with higher levels of C4S in immature six-month-old tissue and higher levels of C6S in mature two year-old tissue (Collin *et al*., 2017). Ovine IVDs also exhibit distinct CS composition in AF and NP with immaturity and maturity (Collin *et al*. 2016). The distinction of young *vs*. old GAG disaccharides in the current study was further identified with PCA analysis as the samples with greatest separation. Collectively with the literature, these data suggested the presence of a unique CS sulphation patern in both NP and AF regions and possibly with age. Thus the use of our GAG analytic method showed potential for identifying changes in CS sulphation unique to ageing, disease and degeneration in the human IVD.

In addition to CS disaccharides, LC-MS also enabled the measurement of HS that, to our knowledge, was the first direct measurement of HS disaccharides in human IVDs. Studies indicate many biological roles for HS, such as that seen in cancer biology (Sasisekharan *et al*., 2002). Specifically, HS disaccharides in urine are shown to be predictive of acute kidney injury and in-hospital mortality in patients with septic shock or acute respiratory distress syndrome (Schmidt *et al*., 2016). While HS disaccharides are not generally reported in IVDs, recent studies suggest a role for certain cell surface HS proteoglycans (*i.e*. syndecan-4 and glypican-6) in AF development, growth and ageing (Becket *et al*., 2015; Binch *et al*., 2016). Our analyses of two predominantly expressed disaccharides in the IVD included that of H0S and H2S. The NP region had similar patterns of H0S and H2S, with the young IVD having greater amounts. In contrast, the AF region of mature IVDs had similar amounts of H0S and H2S while the young IVD had less H2S than H0S. Although these observations were made from a small sample set, the technique enabled distinguishing a strong difference in HS disaccharide patterns between the young and mature specimens, suggesting a possible role in IVD ageing. This potential role could be validated in future studies, using larger sample sizes with wider ranges of age and disease state.

The novel analyses, presented here, generated a large data set quantifying 17 disaccharides on 156 tissue samples providing extensive measurements on IVD GAG types and disaccharides. However, some limitations are important to highlight. These measurements did not determine core proteins, which would require proteomic measurements. This is important, since similar GAG compositions could attach to different proteoglycans to yield distinct functional properties. These analyses did not provide information on disaccharide sequence, quantify KS, or allow distinction between CS and DS, which would all have required additional methodological development. Our study compared mature IVD samples with a single young IVD as a demonstration. This dataset provided estimated differences and variances to perform a power analysis, but did not have sufficient numbers of individual human subjects to statistically test for effects of ageing or degeneration. The results of the current study, along with that available from the literature, point to a potential change in GAG disaccharides with IVD ageing in multiple species; however, it is not clear if there are distinct functional roles for individual GAG disaccharides. GAG disaccharides, core proteins and their sequences may all contribute to functional IVD properties and future studies on their changes with ageing and disease are warranted.

## Conclusion

This study demonstrated the use of a mass spectrometry technique for the quantitative analysis of GAG disaccharides in human IVD samples. We concluded that the high precision and specificity of LC-MS with MRM was capable of quantifying distribution of CS, HA, HS and their disaccharides across various regions of the IVD. The application of PCA further identified regional patterns that were difficult to assess in this large dataset with traditional statistical analyses. Future studies are required to answer how GAG types and their sulphations change with human IVD ageing and degeneration. It is hoped that improved understanding of the roles of GAG types and sulphation patterns may be useful to distinguish ageing from degeneration processes and perhaps inform novel diagnostic techniques or therapies for painful IVD degeneration.

## Figures and Tables

**Table 1:** Specimen, age, sex, disc degeneration grade and cause of death.

Specimen	Age	Sex	Thompson Grade	Cause of Death
**Hu29**	9	M	1	Cardiac arrest
**Hu1**	47	F	2	Respiratory failure
**Hu4**	53	M	3	Cardiac arrest
**Hu18**	62	F	3	Heart disease
**Hu0**	66	F	4	Systolic heart failure
**Hu30**	73	M	3/4	Cancer

**Table 2:** Chondroitin sulphate, heparan sulphate and hyaluronic acid disaccharide structures analysed by LC-MS

	CS disaccharides
TriS_CS_	ΔUA2S(1,3)GalNAc4S6S
2S4S_CS_	ΔUA2S(1,3)GalNAc4S
2S6S_CS_	ΔUA2S(1,3)GalNAc6S
4S6S_CS_	ΔUA(1,3)GalNAc4S6S
2S_CS_	ΔUA2S(1,3)GalNAc
4S_CS_	ΔUA(1,3)GalNAc4S
6S_CS_	ΔUA(1,3)GalNAc6S
0_SC_S	ΔUA(1,3)GalNAc
	**HS disaccharides**
TriS_HS_	ΔUA2S(1,4)GlcNS6S
NS6S_HS_	ΔUA(1,4)GlcNS6S
NS2S_HS_	ΔUA2S(1,4)GlcNS
NS_HS_	ΔUA(1,4)GlcNS
2S6S_HS_	ΔUA2S(1,4)GlcNAc6S
6S_HS_	ΔUA(1,4)GlcNAc6S
2S_HS_	ΔUA2S(1,4)GlcNAc
0S_HS_	ΔUA(1,4)GlcNAc
	**HA disaccharides**
0S_HA_	ΔUA(1,3)GlcNAc

**Table 3:** C6S:C4S ratio within the NP and AF of the young, 9 year old IVD, mean of the mature, 40-80 year old IVD, and mean of all 6 IVD specimens. The C6S:C4S ratio is greater in the mature specimens compared to the young IVD in both the NP and AF.

	young, 9 year (*n* = 1)	mature, 40-80 year (*n* = 5)	mean (*n* = 6)
**NP**	0.44	1.82	1.59
**AF**	0.48	2.22	1.99

**Table 4:** Coefficients of three PCs used to identify IVD regions based on GAG composition from each patient.

	Coefficients of PC1	Coefficients of PC2	Coefficients of PC3
**Hu0 CS**	0.268	0.193	-0.399
**Hu1 CS**	-0.317	0.039	-0.431
**Hu4 CS**	0.386	0.118	-0.053
**Hu18 CS**	-0.178	-0.392	-0.256
**Hu30 CS**	-0.202	0.421	-0.005
**Hu0 HA**	-0.259	-0.204	0.394
**Hu1 HA**	0.323	-0.057	0.425
**Hu4 HA**	-0.386	-0.118	0.064
**Hu18 HA**	0.182	0.405	0.242
**Hu30 HA**	0.186	-0.421	0.004
**Hu0 HS**	-0.245	0.233	0.167
**Hu1 HS**	-0.124	0.317	0.073
**Hu4 HS**	0.165	0.044	-0.353
**Hu18 HS**	-0.045	-0.154	0.173
**Hu30 HS**	0.338	-0.188	0.012

**Table 5:** PCA scores from 28 observations of the inner and outer anterior/posterior/lateral AF regions and NP region used to generate the 3D plots in Fig. 7.

	Scores (PC1)	Scores (PC2)	Scores (PC3)
**AI 1**	3.718	0.534	0.845
**AI 2**	3.885	0.381	-0.267
**AI 3**	3.589	-0.878	-0.923
**AI 4**	4.448	-2.478	-0.156
**LI 1**	0.653	1.449	-0.266
**LI 2**	0.735	1.993	-0.357
**LI 3**	-1.582	2.032	0.633
**LI 4**	1.314	-3.799	0.905
**PI 1**	-2.690	-0.797	1.936
**PI 2**	-0.822	-0.174	2.411
**PI 3**	-1.754	0.000	-0.402
**PI 4**	-0.728	0.689	-0.781
**NP 1**	-3.048	-2.359	-0.433
**NP 2**	-2.378	-1.389	-1.491
**NP 3**	-1.184	-2.245	-1.734
**NP 4**	-1.495	-1.645	-2.348
**AO 1**	0.145	1.622	1.691
**AO 2**	1.125	0.177	1.359
**AO 3**	-1.926	-2.733	1.718
**AO 4**	1.200	0.318	-1.444
**LO 1**	0.100	1.542	-1.248
**LO 2**	-1.019	-0.110	0.993
**LO 3**	-0.425	0.218	1.540
**LO 4**	1.334	0.185	1.202
**PO 1**	-0.773	1.544	-3.677
**PO 2**	-0.337	1.146	-1.204
**PO 3**	-0.325	1.805	0.329
**PO 4**	-1.759	2.972	1.166

## Appendix

### Discussion with reviewers

**Abhay Pandit:** Are there any examples of individual disaccharides of GAGs having a functional role or are all GAG functions related to disaccharide sequences within the GAG?

**Authors:** Multiple studies suggest individual disaccharides play distinct functional roles. For example, chondroitin 4-sulphate (C4S) reduces pro-infammatory markers TNFα, IL-1β and nitric oxide in LPS-treated mouse chondrocytes *in vitro* (Campo *et al*., 2009). Chondroitin 6-sulphate (C6S) disaccharide inhibits neurite growth in embryonic rat neurons (Buterfeld *et al*., 2010). Similarly in the IVD, C6S is abundant in the young notochordal rich-NP tissue and likely contributes to inhibiting neurite growth in rat dorsal root ganglia (known to be involved in innervating IVDs in painful conditions) (Purmessur *et al*., 2015). CS type E from squid cartilage is shown to exhibit potent antiviral activities, specifically against the herpes simplex virus (Bergefall *et al*., 2005). While the exact mechanism of action of these GAG disaccharides is still under investigation, the effects were disaccharide specific and some hypothesise that it is due to the positioning of the sulphate groups. For example, sulphation in carbon position 4 of C4S is opposite a carboxylic group and in the midline of the polymer chain, allowing stronger interactions with structures like nitric oxide, contributing to the anti-infammatory capabilities of C4S. On the other hand, the sulphation at carbon position 6 of C6S occurs more peripherally, thus having a lower affinity for structures like nitric oxide (Campo *et al*., 2009). GAG sulphation paterns also change in tissue development and maturation, suggesting a potential functional role. For example, the chondroitin 4- sulphation / 6- sulphation ratio in embryonic chick brain progressively decreases with development (Kitagawa *et al*., 1997). The chondroitin 4- sulphation /6- sulphation ratio also changes in human synovial fuid and is used as an indicator of joint ageing and osteoarthritis (Nakayama *et al*. 2002). In the IVD, sulphation paterns are altered with ageing in bovine and ovine IVDs (Collin *et al*., 2016; Collin *et al*., 2017). Collectively these data suggest that individual disaccharides of GAGs and their profles play functional roles in the IVD and other tissues.

**Marianna Peroglio:** What diferences can one expect with diferent degrees of degeneration?

**Authors:** Ageing and degeneration results in GAG loss in the NP of IVDs, but GAG loss with degeneration is described as more rapid and/or of larger magnitude than with ageing (*e.g*. Sivan *et al*., 2014; Antoniou *et al*., 1996). However, very few studies investigated the efects of ageing and degeneration on GAG sulphation paterns. Our results suggested an increase in C6S with ageing in human IVDs, and changes in CS sulphation paterns are also observed with maturing and ageing ovine (Collin *et al*., 2016) and bovine IVDs (Collin *et al*., 2017). Our results also exhibited potential age-related shifts in HS sulphation paterns, which are not frequently studied in IVDs. We highlight that ageing and degeneration are distinct processes and hope this method may provide a tool that helps distinguish ageing from degeneration in future studies.

**Marianna Peroglio**: The limits of this methodology also lies in assessment of the glycan structures? How does one determine these?

**Authors:** Our LC-MS method gives important structural information on GAGs, but relies on the removal of GAG chains from core proteins and then the use of chondroitinase ABC. For example, to decompose the CS chains to identify individual CS disaccharides. This precludes us from identifying site-specifc glycan structures, their atachment sites as well as analysis of the core proteins themselves. Methodologies to characterise the glycan structures, along with disaccharide compositions, are only starting to be developed. Glycoproteomic methods such as that developed by Nilsson *et al*. are one recent approach that assesses site-specifc CS proteoglycans and was successfully tested on human urine samples. (Nilsson *et al*., 2017). We previously used 1H-NMR spectroscopy to characterise heparin/HS structure from zebrafsh (Zhang *et al*. 2009). Development of more advanced glycoproteomics methods to advance these techniques would improve knowledge of glycobiology and enable assessments of tissue specifc GAGs in diseased states.

**Peter Roughley**: Do you think the approach used in this study could also be applied to other types of tissues or is it specifc to intervertebral disc?

**Authors:** This approach can be applied to other types of tissues and has been used to successful analyse GAG disaccharides in human urine samples. Using the LC-MS with MRM approach, multiple studies investigating GAG disaccharides in human urine were able to distinguish minute changes in GAG levels from diseased samples such as from patients sufering from mucopolysaccharidoses, acute kidney injury and acute respiratory distress syndrome (Sun *et al*. 2015; Schmidt *et al*. 2016). Our study was one of the frst to validate this technique on human IVD. Given the success of this approach on both body fuid and tissue samples, it seems promising to study other GAG rich tissues such as cartilage and synovial fuid, both of which have shown strong changes in GAG content with development, age and disease.

**Peter Roughley**: How are your fndings impacting the design of repair strategies for IVD?

**Authors:** Improving knowledge of glycobiology in the IVD with ageing and degeneration is a fundamental endeavour without a direct translational pathway. GAG compositional changes, including measurements of disaccharides, are likely to have mechanical and biological roles that may inform the development of more specific tissue engineering constructs or therapeutic targets for IVD repair.

### Additional references

Bergefall K, Trybala E. Johansson M, Uyama T, Naito S, Yamada S, Kitagawa H, Sugahara K, Bergstrom T (2005) Chondroitin sulfate characterized by the E-disaccharide unit is a potent inhibitor of herpes simplex virus infectivity and provides the virus binding sites on gro2C cells. J Biol Chem **280**: 32193-9.

Buterfeld KC, Conovalof A, Caplan M, Panitch A (2010) Chondroitin sulfate-binding peptides block chondroitin 6-sulfate inhibition of cortical neurite growth. Neurosci Let **478**: 82-7.

Campo GM, Avenoso A, Campo S, D'Ascola A, Traina P, Sama D, Calatroni A (2009) Glycosaminoglycans modulate inflammation and apoptosis in LPS-treated chondrocytes. J Cell Biochem **106**: 83-92.

Kitagawa H, Tsutsumi K, Tone Y, Sugahara K (1997) Developmental regulation of sulfation profle of chondroitin sulfate chains in the chicken embryo brain. J Biol Chem **272**: 31377-81.

Nilsson J, Noborn F, Toledo AG, Nasir W, Sihlbom C, Larson G (2017) Characterization of glycan structures of chondroitin sulfate-glycopeptides facilitated by sodium ion-pairing and positive mode LC-MS/MS. J Am Soc Mass Spectrom **28**: 229-241.

Zhang F, Zhang Z, Thistle R, McKeen L, Hosopama S, Toida T, Linhardt RJ, Page-McCaw P (2009) Strucutral characterization of glycosaminglycans from zebrafsh in diferent ages. Glycoconj J **26**: 211-218.

## Abbreviations

IVD: Intervertebral disc
GAG: glycosaminoglycan
CS: chondroitin sulphate
HA: hyaluronic acid
HS: heparan sulphate
AF: annulus fibrosus
AO: outer anterior annulus fibrosus
AI: inner anterior annulus
NP: nucleus pulposus
PI: inner posterior annulus
PO: outer posterior annulus
LI: inner posterior-lateral annulus
LO: outer posterior-lateral annulus
PCA: principal component analysis
PC: principal component
MRM: multiple reaction monitoring
LC-MS: liquid chromatography-mass spectrometry
